# Foreign Correspondence — Letter from Paris

**Published:** 1868-04-15

**Authors:** 


### *FOREIGN CORRESPONDENCE.*

PARIS, *February 7th*, 1868.

*Chicago Medical Journal:*

Many years ago, M. Gillermond published a process for obtaining *Morphine*, then recently discovered, from an alcoholic solution of *Opium*, by means of *Ammonia.* It being rather a costly process, it was not adopted; but in 1849, the son of the discoverer, Dr. Gillermond, of Lyons, showed that it possessed some advantages in the testing of the quantity of *Opium.* Some difficulties in the preparation have now induced the same practitioner to publish an explanatory note on this subject. The process is as follows: Dissolve 15 grammes of *Opium*, by degrees, in 100 grammes of *Alcohol*, and strain the tinctures successively through a cloth into a wide-necked phial, containing 4 grammes of *Ammonia.* After the lapse of twelve hours, the *Morphine* will be found eliminated with a certain quantity of *Narcotine*; the former having coated the glass with colored crystals, large enough to be gravelly to the touch, while the *Narcotine* has crystallized in small white needles, having the appearance of mother-of-pearl. The whole of the crystals are collected on a piece of linen, and well washed with water, in order to deprive them of the *Meconate of ammonia* they may happen to contain. The crystals are taken up again, and thrown into a small receiver full of water. The *Narcotine*, which is lighter than the fluid, will float at the top; the *Morphine*, which is heavier, will fall to the bottom; and the former substance may be separated from the latter by decantation. A good sample of *Opium* should yield at least from 1 gramme 25 centigrammes, to 1 gramme 50 centigrammes of crystallized *Morphine*, for every 15 grammes of *Opium.* The separation of the *Narcotine* from the *Morphine* may not always be complete by this process; but it may be, if necessary, effected by repeatedly pouring boiling *Ether* on. Dr. Gillermond has improved his process by triturating 15 grammes of *Opium* with 110 grammes of *Alcohol*, at 70-100th's, or 120 cubic centimeters. When the dissolution is complete, which generally takes place in half an hour, the weight of 125 grammes, if diminished, should be made up with *Alcohol;* the solution is then well shaken, filtered into a phial, and a tube, thinned off at one end, being introduced, 2 grammes of *Ammonia* are poured in, so as to reach the bottom without disturbing the liquid. The tube is then quietly removed, and the phial closed; and after standing thirty-six hours, the *Morphine* will be found crystallized at the bottom of the phial.

Were all the cures proposed for hydrophobia, up to this day, collected into a volume, it might equal the largest ever brought to light. But, compared with the vast number of devices put forward for curing the disease when actually caught, suggestions of a preventive nature have been exceedingly few; and this circumstance constitutes the chief merit of an article in the *Tiglo Medical*, a Spanish medical paper. For a long time past, this journal informs us, the peasants of Gallicia have entertained the idea that dogs bitten by vipers, which are very common in that province, are no longer liable to contract hydrophobia, whether spontaneous or inoculated, after recovering from the bite of the reptile. This belief has induced them to endeavor to protect their dogs from the danger of *rabies canina*, by having them bit by vipers when young. If, indeed, the venom of the viper be a preservation against the other virus, the inoculation of the former will, by preventing dogs from contracting hydrophobia, indirectly protect men from being attacked with it. This consideration has induced a Spanish physician, whose name, unfortunately, is not given, to institute experiments with a view to test the efficacy of this preservation, by causing a number of young dogs, five or six months old, to be stung by vipers; and after recovery, to be subjected to the bite of mad dogs. The result was, that in every case the former were found to be insensible to the virus of hydrophobia, no matter how often or at what period of their lives they were subjected to the ordeal. The sting of the viper would bring on fever, somnolency and uneasiness, increasing for three or four days, and then subsiding in the course of three or four days more. Olive oil, externally and internally administered, would afford some relief. If, five or six months later, the same dog were stung a second time, the only consequence would be a slight tumefaction, without fever; and any subsequent bite by a viper produces no effect whatever. Without venturing to contradict the facts here stated, it is difficult to imagine how they can be reconciled with the process of elimination, which invariably takes place when a poison, introduced into the economy, has not occasioned death. This subject is attracting considerable attention at the Academy of Medicine, and experiments are being instituted to ascertain the value of the remedy proposed.

The *Journal des Connaissances Médicale* notices a book recently published by Dr. Foissac, on the influence of climate and physical agents on man. The author maintains that the human race is cosmopolitan, since it can live every where, and by its intellectual powers neutralize the evil effects of physical agents on its organism. To this, Dr. Caffes demurs, objecting that man does not perpetuate his race under all climates; that he may, it is true, in any climate to which he is taken in the prime of life, but that sterility is often the consequence, and that, at any rate, his offspring will die at an early age. However this may be, Dr. Foissac's book contains much interesting matter; and the chapter on stature comprises a great many new and valuable facts. On this subject, Dr. Latour, in his review of the volume, expresses himself as follows: "No one will maintain that good soldiers are not to be found among small men. During the campaign in Egypt, Moored Bey's vexation would break out whenever he made a few of our brave *voltigeurs* prisoners. 'What!' he would exclaim, 'are these the men that have beaten us? Shall I never be able to vanquish these little fellows?'". Yet, Dr. Foissac maintains, on the strength of highly reliable historical records, that the inhabitants of ancient Gaul, who were victors and conquerors by turns, but always terrible on the field of battle, were tall and fine men—contrary to Dr. Broca's opinion. To the low or middling stature of Alexander, Napoleon, and Gustavus Adolphus, he opposes the gigantic proportions of Philopœmus, Pyrrhus, Cæsar, Charlemagne, Condé, Peter the Great, and Charles the Twelfth. Most of the generals of the Republic and marshals of the First Empire, such as Championnet, Kleber, Pichegru, Masséna, Soult, Remadotte, Kellerman, Bessiéres, and Ney, were very tall—or, at least, much above the common standard. Dr. Foissac finds the latter condition not only in the case of military men of note, but also in that of great political characters—orators, poets, learned men, and, generally, of most men representing intellectual power; whence he concludes that, save in the case of deformity, genius and talent are independent of physical conformation. Scarron and Pope would seem to nullify even the above restricted clause.

The death is announced, at the age of 92, of M. Carlo Speranza, the Nestor of Italian physicians, formerly Dean of the Faculty of Medicine and University of Pavia. M. Speranza was author of several important works, and was, if I mistake not, corresponding member of the Academy of Medicine in this city.

The *Clinique Européenne* contains the following account of certain experiments made by M.M. Klautoch and Stich, to ascertain the real seat of the sense of taste, which is generally supposed to exist on the whole surface of the tongue, especially the anterior part of that organ, the middle of the dorsum being but feebly endowed with this sense. It seems, from these experiments, that the only portion of the tongue which is sensible to taste, is a narrow space all around. The breadth of this sensitive zone varies in different subjects; in some, it is not more than two lines; in others double that breadth. It rarely extends to the inferior surface. The experiments were as follows: A substance having a strong taste, is first placed on the centre of the tongue, where it produces no effect. It is then gradually spread out, until the perception of taste is announced; this occurs generally on the border, but in some individuals it begins at the distance of a line from it. The *velum pendulum* of the palate is also sensible to taste; but the pharynx and the tonsils are deprived of the gustatory faculty. This is proved by the fact that if they be touched with stick caustic the patient experiences no taste, provided he keep the tongue and the velum pendulum away from the spot.

The *Abeille Médicale*, in speaking of those soft and elastic dropsical tumors of the skin, called anasarcas, states that *Tannin* has been successfully applied by Dr. Garnier against them. This substance, the astringent principles of galls and other bitter vegetable substances, is administered in solution, in a dose of two grammes per thirty grammes of syrup, diluted with the same quantity of distilled water. Three tablespoonsful per day are given, and the dose gradually increased according to the effect obtained. The *Abeille* quotes three cases in which an almost immediate cure was obtained by this method.

In a paper published by the *Reveue Thérapeutique*, Dr. Ozanam treats of substances capable of destroying the adventitious membrane in croup and malignant quinsy. From his experiments and observations, it appears that the best substances for dissolving the membrane are, first of all, *Ammoniuret of copper*, then *Ammonia*, *Soda*, *Urea*, *Cyanide of potassium*, *Chloride of potassium*, *Glycerine*, *Lime water*, and common *Salt*. *Carbonate* or *Chlorate of potash*, and *Phosphate of soda*, are of but slight efficacy. The dissolving agents of great power are: *Chloride of bromine*, *Bromine* itself, and *Chlorine*; then, secondary to these, *Iodine*, *Perchloride of iron*, *Bichloride of mercury*, and *Chromium*, which harden the adventitious membrane, and detach it in a mass, without severing its elements. Dr. Ozanam is of opinion that sea-water, which contains *Bromine* and the elements of the mother-ley of *Soda*, extracted from *Wrack*, is an excellent remedy in all cases of bad sore-throat; but he prefers *Bromine* to all others, not only as a powerful dissolvent, but also because it exerts an elective action on the fauces, the velum pendulum, palate, and the larynx. *Bromine* possesses another valuable property: like *Chlorine*, it destroys contagion, besides being more manageable. With its emanations, the air may be purified, the dormitories and whole houses protected from contagion, at an insignificant expense. But as it is a most powerful agent, it can not be used in a pure state. It should not be employed under the forms of a tincture, because new compounds are then formed; nor should it be administered in tea, for it would then fix itself upon the vegetable principle, and lose all action. The way to use it is to make an aqueous solution, in the proportion of a drop of *Bromine* to an ounce of water, to be kept in a dark place, and in a closed phial. This solution must be renewed as soon as it loses its amber hue. It is administered in drops every hour, and taken in spoonsful of sugar and water, so as to give from one to two grammes of the solution in the course of twenty-four hours.

M. Pouchet combats the opinion of some physiologists who are willing to admit that certain animals may survive after being completely frozen. From numerous experiments made on several species of animals, he comes to the conclusion that there always takes place, in the congealed part, besides concontraction of the capillary vessels, an alteration of the blood globules, characterized by the disappearance of the nucleus in the reptiles, and by the appearance of crenelated projections in mammalians. Thus altered, the blood globules can not fulfill their functions, and the life of the animal is all the more in jeopardy, that the number of altered globules flowing in the current of the circulation is large. If a portion of the body of an animal be congealed, and maintained in that state, the animal may live a long while yet, owing to the altered blood being unable to mix with the circulatory current, on account of its having lost its fluidity; but death soon occurs, after the frozen parts are thawed out; the altered globules mixing, in great quantity, with the remaining portion of the sanguineous fluid. In every case of congelation, death is due to the altered state of the blood, and not to the stupefied condition of the nervous system.

The discussion on tuberclosis is still going on, at the Academy of Medicine. No new facts have been elicited on that subject, although several ingenious and plausible theories have been brought forward. In my next letter, I will give you an outline of the debates held on that obscure though most important subject. N.

